# High levels of gene flow and genetic diversity in Irish populations of *Salix caprea* L. inferred from chloroplast and nuclear SSR markers

**DOI:** 10.1186/s12870-014-0202-x

**Published:** 2014-08-07

**Authors:** Aude C Perdereau, Colin T Kelleher, Gerry C Douglas, Trevor R Hodkinson

**Affiliations:** Teagasc, Agriculture and Food Development Authority, Kinsealy Research Centre, Malahide Road, Dublin, D17 Ireland; Botany Building, School of Natural Sciences, Trinity College Dublin, Dublin, D2 Ireland; Trinity Centre for Biodiversity Research, Trinity College Dublin, Dublin, D2 Ireland; DBN Plant Molecular Laboratory, National Botanic Gardens, Glasnevin, Dublin, D9 Ireland

**Keywords:** Genetic diversity, Microsatellites, Population structure, *Salix*, Willow

## Abstract

**Background:**

*Salix caprea* is a cold-tolerant pioneer species that is ecologically important in Europe and western and central Asia. However, little data is available on its population genetic structure and molecular ecology. We describe the levels of geographic population genetic structure in natural Irish populations of *S. caprea* and determine the extent of gene flow and sexual reproduction using both chloroplast and nuclear simple sequence repeats (SSRs).

**Results:**

A total of 183 individuals from 21 semi-natural woodlands were collected and genotyped. Gene diversity across populations was high for the chloroplast SSRs (*H*_*T*_ = 0.21-0.58) and 79 different haplotypes were discovered, among them 48% were unique to a single individual. Genetic differentiation of populations was found to be between moderate and high (mean *G*_*ST*_ = 0.38). For the nuclear SSRs, *G*_*ST*_ was low at 0.07 and observed heterozygosity across populations was high (*H*_*O*_ = 0.32-0.51); only 9.8% of the genotypes discovered were present in two or more individuals. For both types of markers, AMOVA showed that most of the variation was within populations. Minor geographic pattern was confirmed by a Bayesian clustering analysis. Gene flow via pollen was found to be approximately 7 times more important than via seeds.

**Conclusions:**

The data are consistent with outbreeding and indicate that there are no significant barriers for gene flow within Ireland over large geographic distances. Both pollen-mediated and seed-mediated gene flow were found to be high, with some of the populations being more than 200 km apart from each other. These findings could simply be due to human intervention through seed trade or accidental transportation of both seeds and pollen. These results are of value to breeders wishing to exploit natural genetic variation and foresters having to choose planting material.

## Background

The genus *Salix* L. (willows, sallows and osiers) belongs to a family of catkin-bearing trees, the Salicaceae. The basic chromosome number of *Salix* is 19, and most species are diploid (2x = 38), but ploidy levels up to dodecaploid (12x = 228) have been reported [1]. Most willows can be easily propagated from hardwood cuttings, although some species are not good rooters e.g., *S. caprea* L. and *S. scouleriana* Barratt [2,3]. *Salix* flowers are predominantly insect-pollinated, but wind-pollination also occurs [4].

Microsatellite markers have been developed successfully for some species of willows. They have been characterized in *Salix burjatica* Nasarow [5], *S. reinii* Franch. & L. Sav. [6], subarctic willows, *S. lanata* L., *S. lapponum* L. and *S. herbacea* L. [7], *S. hukaoana* Kimura [8], *S. arbutifolia* Pall. [9] and up to 31 different species of willows in Barker et al. (2003) [10]. A subset of markers from this later publication have been tested and applied in this paper. SSR markers were used as they are co-dominant and allow data comparison between different studies. A high level of polymorphism makes them suitable for inferring relatively recent population genetic events; they can also be used to genetically discriminate between individuals and populations [11].

*Salix caprea* is a cold-tolerant pioneer species native to Ireland which occurs in a broad range of habitats and is one of the few willow species able to grow in forest understories [12]. It is frequently found growing in hedgerows, by woodland margins or on rocky lake shores as it is more tolerant of dry situations than many other willows. It also colonises disturbed sites and waste ground [13]. It is sometimes used in breeding programmes for short rotation coppice cultivars [14]. Only one population genetic study has examined natural populations of *S. caprea* [15], which studied four PCR-RFLP markers and three chloroplast SSRs on 24 European populations. High levels of variation within populations were detected and no distinct phylogeographic structure was revealed among populations at the European scale. No studies have examined genetic variation in Irish *S. caprea*.

However, the molecular ecology of several other species of *Salix* has been studied throughout the world. Lian et al. (2003) [16] used nuclear and chloroplast microsatellites to examine population genetic structure and reproduction dynamics in *S. reinii*, a creeping shrub which is a pioneer colonist of volcanic substrates on Mount Fuji, Japan. Evidence of clonal growth and seedling recruitment were detected in this polyploid species.

A study has been conducted in the UK for conservation and restoration of *S. lanata* and *S. lapponum* [17]. They found distinct multi-locus genotypes for most individuals with five SSR markers, and were able to deduce that sexual reproduction is the predominant means of perpetuation and dispersal at the site of study. However, they also examined a more common subarctic willow (*S. herbacea*) and found evidence of clonal growth in individuals growing up to seven metres apart.

Another study in the USA focused on a native willow (*S. eriocephala* Michx.) and a naturalized one (*S. purpurea* L.) to compare the genetic diversity and structure of their populations [18]. Their results revealed that some subpopulations of *S. purpurea* contained plants with identical multilocus genotypes (inferred to be clones), while clonal individuals were rare among *S. eriocephala* populations. They suggest that vegetative propagation in combination with sexual reproduction has contributed to the naturalization of *S. purpurea* in the USA and has resulted in higher levels of genetic differentiation among *S. purpurea* populations than among native *S. eriocephala* populations [18].

Population genetic structure was recently studied in the endangered *Salix daphnoides* Vill. in the Czech Republic [19]. 174 individuals from 14 populations were analysed using SSR and AFLP markers. High genotypic variability and heterozygosity were revealed with the SSR markers in the natural populations.

In order to investigate the genetic diversity, the extent of gene flow and the population genetic structure of natural Irish populations of *S. caprea*, we analysed nuclear and chloroplast microsatellite markers. A combination of statistics were applied including 1) traditional population-genetic methods that often require *a priori* population designation such as diversity statistics, allele frequencies across Ireland, unique genotypes, analysis of variance, and tests of isolation by distance, and 2) Bayesian algorithms that cluster individual samples into populations without *a priori* population designation. Results were compared to those presented in previous studies on other woody species with a particular focus on Salicaceae.

The specific aims were to test existing chloroplast and nuclear SSR markers for their ability to detect and describe genetic diversity and differentiation of populations in *S. caprea*, describe nuclear and cpDNA allelic and haplotypic diversity in natural Irish populations of *S. caprea*, determine the level of geographic population genetic structure in natural Irish populations of *S. caprea*, and determine the extent of gene flow and sexual reproduction in this species.

## Methods

### Sample collection

*Salix caprea* was sampled in semi-natural woodlands, defined hereafter as “woodlands which resemble the natural woodland cover, dominated by native trees but altered by human activity. Stands originating from previous planting may be termed semi-natural if they are now regenerating naturally, as may stands which were formerly coppiced” [20]. Ireland is one of the least wooded countries in Europe with approximately 10% of land covered by forests. However 80,000 hectares or about one percent of Ireland’s land area is native woodland with the rest being non-native coniferous trees [21]. In order to find sites suitable for study, the herbarium specimens in Trinity College Dublin, Ireland and in the National Botanic Gardens, Dublin, Ireland were examined for site location information. The native woodland survey database [22] was also checked.

**Figure 1:**
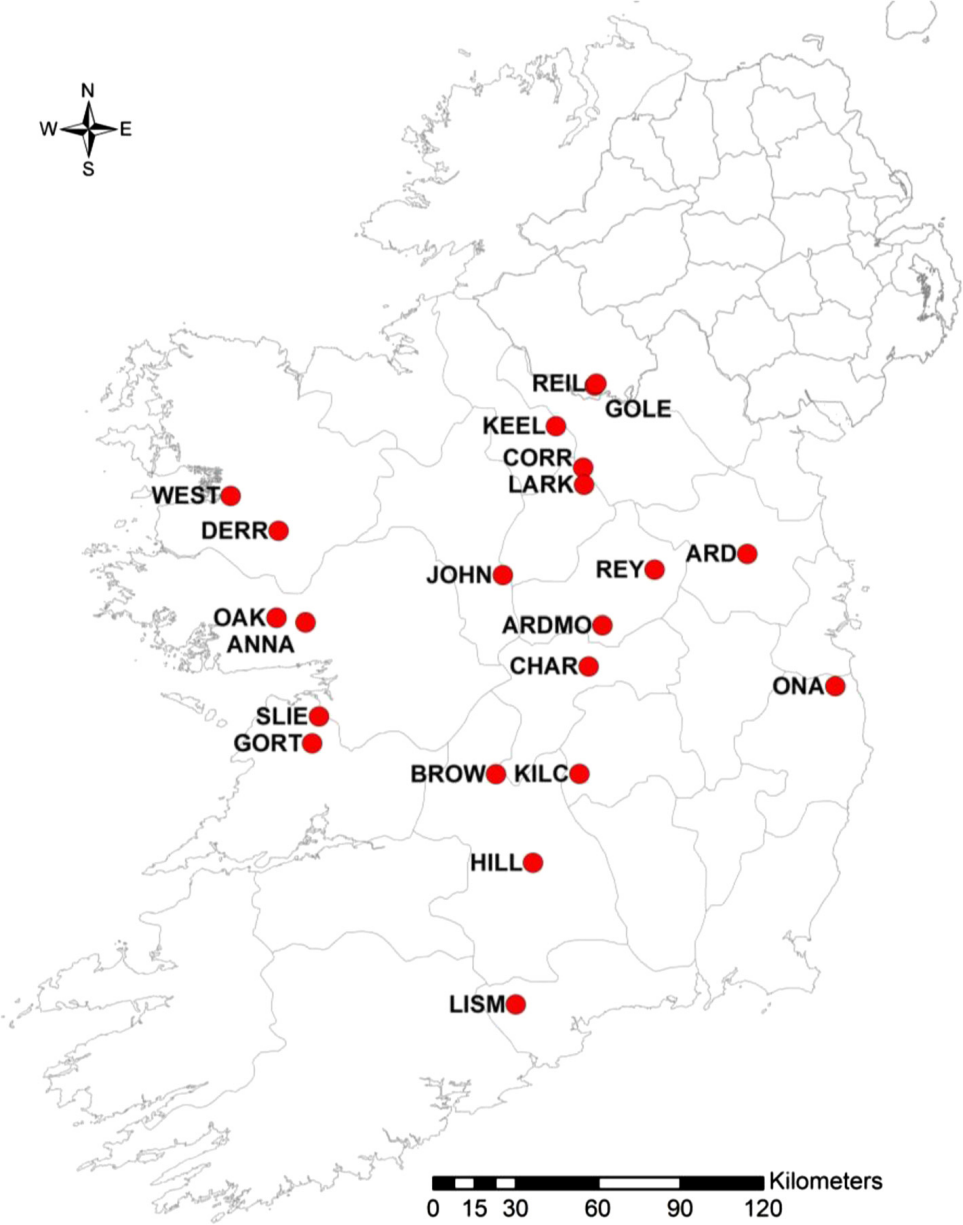
**Sites for the natural populations of** ***S. caprea.***

Samples of leaves of natural populations of *Salix caprea* were collected across Ireland during the summers of 2010 and 2011. 183 individuals from 21 sites in counties Cavan, Clare, Fermanagh, Galway, Laois, Leitrim, Longford, Mayo, Meath, Offaly, Roscommon, Tipperary, Waterford, Westmeath and Wicklow were sampled (Figure 1). Between 7 and 23 individuals were collected per site (Table 1). A few young green leaves were taken from each tree and stored in silica gel [23]. The distinction of *Salix caprea* from other willows is relatively clear. For correct identification in the field, Meikle, 1984 [13] and Webb et al., 1996 [24] were used.

**Table 1:** **List of the collection sites, code and number of samples analysed**

**Location**	**Code**	**Number of samples**
Ardsallagh Co. Meath	ARD	8
Onagh Co. Wicklow	ONA	8
Reynella house Co. Westmeath	REY	23
Ardmorney Co. Westmeath	ARDMO	8
Larkfield Co. Longford	LARK	8
Corratober Co. Cavan	CORR	8
Keelrin Co. Leitrim	KEEL	8
Brownstown Co. Offaly	BROW	8
Kilcoke Co. Laois	KILC	8
Charleville Co. Offaly	CHAR	8
Annaghdown Co. Galway	ANNA	8
Slievecarron Co. Clare	SLIE	8
Gortlecka Co. Clare	GORT	8
St John’s wood Co. Roscommon	JOHN	8
Oakfield Co. Galway	OAK	8
Westport Co. Mayo	WEST	8
Derrinrush Co. Mayo	DERR	8
Gole wood co. Fermanagh	GOLE	7
Reilly wood co. Fermanagh	REIL	9
Lismore co. Waterford	LISM	8
Killough hill co. Tipperary	HILL	8

### Amplification and genotyping

DNA was extracted from dried leaf tissue with a DNeasy Plant Extraction kit (Qiagen, Valencia, CA, USA). The markers used included eight chloroplast and six nuclear SSR loci. The chloroplast SSR markers were produced using a set of primers designed for universal application for dicotyledonous angiosperms and were developed on tobacco (*Nicotiana tabacum* L.) [25]. CCMP2, 3, 4, 5, 6, 7, 8 and 10 were used. They are located mostly in intron and intergenic regions. The nuclear markers were designed specifically for *Salix* spp. from an enriched library of *Salix burjatica* [10]. SB24, 38, 85, 93, 194 and 199 were used. Loci were genotyped with automated capillary based electrophoresis and fluorescently labelled primers. Each forward primer of a pair was labelled on the 5’ end with a fluorescent dye (JOE™ TAMRA or 5-FAM™).

Prior to amplification by PCR, the quantity of DNA of each sample was checked using a NanoDrop 2000 spectrophotometer (Thermo Scientific). Amplification using the CCMP primers was as follows (12.5 μL total volume): 10 ng DNA, 1× colorless GoTaq® Flexi Buffer, 0.2 mM of each dNTP, 0.2 μM of each primer, 1.5 mM MgCl_2_, 0.25 units of GoTaq® DNA Polymerase. Every primer was used at 0.2 μM except forward and reverse primers of CCMP5 which were both used at 0.4 μM. PCR parameters included 95°C for 4 min, then 35 cycles at 95°C for 30 s, 50°C for 45 s and 72°C for 1 min 15 s, following a final extension at 72°C for 8 min. Amplification using the nuclear SB primers (12.5 μL total volume) used 10 ng DNA, 1× colorless GoTaq® Flexi Buffer, 0.2 mM of each dNTP, 0.32 μM of each primer, 1.5 mM MgCl_2_, 0.25 units of GoTaq® DNA Polymerase. SB38 and SB85 forward and reverse primers were both at 0.4 μM whereas the others were at 0.32 μM. The PCR parameters were 94°C for 2 min, then 35 cycles at 94°C for 40 s, 54°C for 1 min and 72°C for 2 min, following a final extension at 72°C for 20 min. The annealing temperature was different depending on the primers, it was 48°C for SB38, 50°C for SB85, 52°C for SB194 and 54°C for SB24, SB93 and SB199.

Between 1:5 and 1:80 dilutions were performed according to the brightness of the band after checking the quantity of DNA on an agarose gel. PCR products were multiplexed and 1 μL of the diluted mix was added to 8.75 μL Hi-Di formamide and 0.25 μL of an internal lane size standard (Genescan™ 400HD-ROX Standard; Applied Biosystems) and run on an ABI 3130xl Genetic Analyzer (Applied Biosystems), following the manufacturer’s protocol. After genotyping, the fragments were sized using GeneMapper v4.1 (Applied Biosystems).

### Data analyses

For both the chloroplast and nuclear markers, the frequency distribution of each marker was graphed in Microsoft Excel and mapped into ArcGIS 10.1 (ESRI) for each population. Diversity indicators were calculated in total, per population and per locus in GenAlEx 6.5 [26], POPGENE 1.31 [27] or Arlequin 3.1 [28]. Number of different alleles, number of effective alleles (1/(Σ*p*_*i*_^2^)), Shannon's information index (−1*Σ(*p*_i_*ln(*p*_*i*_))), gene diversity/expected heterozygosity [29] (1-Σ*p*_*i*_^2^), (where *p*_*i*_ is the frequency of the i^th^ allele and Σ*p*_*i*_^2^ is the sum of the squared allele frequencies), observed heterozygosity (number of heterozygotes/n) and Jost’s estimate of differentiation [30,31] were calculated in GenAlEx [32].

POPGENE was used to calculate overall diversity in collections (total gene diversity = *H*_*T*_), diversity within populations (*H*_*S*_), genetic differentiation (*G*_*ST*_ = 1-*H*_*S*_/*H*_*T*_), inbreeding coefficient within individuals in each subpopulation (*F*_*IS*_), inbreeding coefficient of an individual relative to the total population (*F*_*IT*_), inbreeding coefficient within subpopulations, relative to total (genetic differentiation among populations, *F*_*ST*_ = (*H*_*T*_-*H*_*S*_)/*H*_*T*_). The values of *G*_*ST*_ were taken to calculate the ratio of pollen mediated/seed mediated gene flow [33].

The average gene diversity over loci was calculated in Arlequin. Exact tests of Hardy-Weinberg (HW) equilibrium using a Markov chain were performed in Arlequin for the nuclear loci. Analyses of molecular variance (AMOVA) were carried out in Arlequin with two different analyses of distance, the number of different alleles (*F*_*ST*_) based on the infinite allele model and the sum of squared size difference (*R*_*ST*_) based on the stepwise mutation model. Gene flow was estimated from *F*_*ST*_ obtained from the AMOVAs (*Nm* = (1-*F*_*ST*_)/*F*_*ST*_ for cpSSR data or *Nm* = 0.25*(1-*F*_*ST*_)/*F*_*ST*_ for the nuclear SSR data) [34]. Unique multilocus genotypes per population and in total were inferred using GeneticStudio [35].

Isolation by distance (IBD) estimation was carried out using a Mantel test. Two types of test were made: 1) with all the individuals against the haploid genetic distances matrix (for the cpSSRs) or the codominant genotypic distances matrix (for the nuclear markers) obtained from GenAlEx, or 2) with the matrix of Slatkin linearized *F*_*ST*_ for each population obtained from Arlequin [36]. Both tests were performed in GenAlEx with 9999 permutations.

Genetic structure was investigated using STRUCTURE v2.3.4. [37,38], which applies the Markov Chain Monte Carlo (MCMC) algorithm. This procedure clusters individuals into populations and estimates the proportion of membership in each population for each individual. An admixture model with correlated allele frequencies was used, the *K* value was set from one to ten, and ten runs were performed for each value of *K*. The length of the burn-in period was set to 50,000, and the MCMC chains after burn-in were run for an additional 100,000 times. The optimal value of *K* was determined by examination of the Δ*K* statistic [39] using Structure Harvester [40].

## Results

### Overall frequencies of the alleles detected

#### cpSSRs

A maximum of one allele per locus per individual was detected. They were the predicted length suggesting there was no contamination and the target region was amplified. 31 alleles were discovered in total for the 8 cpSSRs. Microsatellites CCMP4 and CCMP7 were found to be monomorphic (respectively 113 and 133 bp) but the other microsatellites were variable, with mononucleotide repeats in every case (1 bp difference). Between 2 and 7 alleles per locus were found (Table 2). CCMP5 was the most variable, and CCMP10 was the least variable, with only 2 size variants (102 and 103 bp). No obvious geographical patterns were detected when the allele proportions at each microsatellite locus were mapped per population (data not shown).

**Table 2:** **Diversity indicators for the different chloroplast SSR markers across all populations**

**Locus**	***N***	**Pred**	**Size**	***N***_***A***_	***N***_***E***_	***I***	***H***_***T***_	***H***_***S***_	***G***_***ST***_
CCMP2	170	189	210-215	6	3.59	1.48	0.73	0.45	0.38
CCMP3	183	112	102-104	3	2.15	0.92	0.48	0.28	0.42
CCMP5	180	121	101-107	7	2.90	1.32	0.66	0.43	0.35
CCMP6	179	103	108-116	6	3.66	1.42	0.73	0.47	0.35
CCMP8	183	77	69-73	5	2.85	1.22	0.65	0.44	0.32
CCMP10	181	103	102-103	2	1.15	0.26	0.14	0.02	0.84
CCMP4	155	126	113	1					
CCMP7	179	133	133	1					
Mean	176			4.8	3.59	1.48	0.57	0.35	0.38
SE	1.98			0.8	2.15	0.92	0.05	0.03	

*N* = Sample Size, Pred = Predicted product size (bp) from Weising and Gardner [25] in tobacco, Size = Allele size range (bp), *N*_*A*_ = No. of Obtained Alleles, *N*_*E*_ = Effective No. of Alleles, *I* = Shannon's Information Index, *H*_*T*_ = Diversity in overall collections total gene diversity, *H*_*S*_ = Diversity within populations, *G*_*ST*_ = Genetic differentiation.

#### Nuclear SSRs

All six nuclear markers were found to be polymorphic with between 3 and 16 alleles per locus (mean = 10) (Table 3). A total of 60 alleles were detected from the 6 markers. The repeats were di- or tri-nucleotide in every case. A maximum of two alleles per locus per individual was detected and this is consistent with the expectation that all plants were diploids. SB24 was the most variable with 16 alleles and SB85 was the least variable with only 3 alleles (Table 3). No obvious geographical patterns were detected when the allele proportions at each microsatellite locus were mapped per population (data not shown).

**Table 3:** **Diversity indicators obtained from the nuclear SSR markers**

**Locus**	***N***	**Pred**	**Size**	***N***_***A***_	***N***_***E***_	***I***	***H***_***O***_	***H***_***E***_	***HW***	***F***_***IS***_	***F***_***IT***_	***F***_***ST***_	***G***_***ST***_	***D***_***est***_
SB24	362	109-245	124-182	16	2.8	1.57	0.62	0.64	NS	−0.09^NS^	0.03	0.11**	0.05**	0.09**
SB38	364	105-161	106-156	15	9.0	2.34	0.86	0.89	NS	−0.09^NS^	0.03	0.11**	0.06**	0.33**
SB85	362	81-87	79-85	3	1.1	0.11	0.04	0.04	NS	−0.12^NS^	−0.02	0.09*	0.04*	0.002*
SB93	336	159-185	150-168	7	2.1	0.92	0.07	0.53	S	0.85^NS^	0.88	0.23*	0.12*	0.13*
SB194	362	105-152	108-130	11	5.9	1.95	0.75	0.83	S	−0.04^NS^	0.09	0.13**	0.07**	0.27**
SB199	212	102-140	98-126	8	2.4	1.20	0.10	0.58	S	0.71^NS^	0.79	0.28*	/	/
Mean^1^	333			10	3.9	1.35	0.41	0.58		0.08^NS^	0.30	0.16**	0.07**	0.10**
SE	24.6			2	1.2	0.32	0.15	0.12						

*N* = Sample Size, Pred = Predicted product size (bp) from Barker [10], Size = Allele size range (bp), *N*_*A*_ = No. of Obtained Alleles, *N*_*E*_ = No. of Effective Alleles, *I* = Shannon's Information Index, *H*_*O*_ = Observed Heterozygosity, *H*_*E*_ = Expected Heterozygosity, *HW* = exact test of Hardy-Weinberg equilibrium with a significance at *p* = 0.01, *F*_*IS*_ = Inbreeding coefficient within individuals in each subpopulation, *F*_*IT*_ = Inbreeding coefficient of an individual relative to the total population, *F*_*ST*_ = Genetic differentiation among populations, *G*_*ST*_ = Analog of *F*_*ST*_, *D*_*est*_ = Jost’s estimate of differentiation. ^1^Mean over loci rather than the arithmetic average, ^NS^non significant, *P < 0.05; **P < 0.001 Probability values are based on 999 permutations.

### Genetic diversity

#### cpSSRs

Indicators of genetic diversity are provided in Table 2. The number of effective alleles (*N*_*E*_) ranged from 1.15 for CCMP10 to 3.66 for CCMP6. *H*_*T*_ ranged from 0.13 for CCMP10 to 0.73 for CCMP6. *H*_*S*_ was lower than the overall diversity for all markers, as not all the alleles were present in every population. *Nm* was equal to 2.29, *Nm* > 1.0, which shows little differentiation among populations.

#### Nuclear SSRs

The number of effective nuclear alleles was lower than the total number of alleles, showing that few alleles contributed to the variation (Table 3). Average heterozygosities (*H*_*E*_) were variable across loci reflecting the different number and frequencies of the alleles found. For three loci, *HW* tests were significant. It was especially visible for SB93 and SB199 where a small *H*_*O*_ was observed, this was confirmed by both *F*_*IS*_ and *F*_*IT*_ where these indicators were found to be high, indicating a dearth of heterozygotes at these two loci. No excess of heterozygotes were detected even for the SB194 loci. *Nm* was equal to 2.76, *Nm* > 1.0 again, which indicates a constant gene flow among populations.

#### Analysis per population

A mean number of 2.2 alleles and 3.9 alleles were found per locus and per population for the chloroplast and nuclear SSRs respectively (Table 4). The average gene diversity over all samples was high and was similar for both types of markers (0.56), showing that two randomly chosen genes will carry different alleles roughly half of the time. For each population, the observed heterozygosities (*H*_*O*_) were less than the expected heterozygosities (*H*_*E*_) except for CORR and KILC where an excess of heterozygotes was observed.

**Table 4:** **Diversity indicators per population obtained from the chloroplast and nuclear SSR markers**

		**cpSSRs**			**Nuclear SSRs**					
**Sampling site**	***N***	***UH***	***A***	***H***	***UG***	***A***	***H***	***H***_***E***_	***H***_***O***_	***P***
ANNA	8	63	2.2	0.41	100	4.5	0.59	0.56	0.51	83.3
ARD	8	88	2.3	0.43	100	3.8	0.49	0.48	0.39	83.3
ARDMO	8	63	2.7	0.53	100	4.2	0.60	0.52	0.35	83.3
BROW	8	75	2.3	0.46	100	3.3	0.48	0.46	0.46	66.7
CHAR	8	75	2.3	0.42	100	4.2	0.52	0.48	0.39	83.3
CORR	8	63	2.2	0.32	37.5	2.8	0.37	0.35	0.48	66.7
DERR	8	75	2.5	0.42	100	5.3	0.58	0.58	0.46	100
GOLE	7	71	2.0	0.31	100	4.2	0.62	0.54	0.51	100
GORT	8	75	2.3	0.54	100	3.8	0.47	0.45	0.32	83.3
HILL	8	75	1.8	0.34	100	3.3	0.52	0.48	0.40	83.3
JOHN	8	63	2.0	0.27	100	4.0	0.53	0.51	0.40	83.3
KEEL	8	88	3.0	0.58	100	3.7	0.56	0.53	0.42	83.3
KILC	8	88	2.5	0.45	100	4.0	0.50	0.41	0.44	83.3
LARK	8	75	2.2	0.40	100	4.5	0.56	0.51	0.39	100
LISM	8	50	1.5	0.24	100	4.0	0.56	0.57	0.43	100
OAK	8	50	2.2	0.27	100	3.8	0.55	0.51	0.40	83.3
ONA	8	88	2.8	0.49	100	4.7	0.61	0.56	0.44	83.3
REIL	9	67	1.8	0.29	100	3.8	0.58	0.53	0.41	83.3
REY	23	43	2.5	0.47	100	4.5	0.51	0.50	0.37	83.3
SLIE	8	50	1.7	0.21	100	2.8	0.42	0.39	0.38	66.7
WEST	8	50	1.8	0.34	62.5	2.7	0.45	0.45	0.33	83.3
Average over populations		68	2.2	0.39	95.6	3.9	0.53	0.49	0.41	84.1
Average*	8.7	48.1	4.8	0.56	90.2	10.2	0.56	0.59	0.41	100

*N* = Number of samples per site, *UH* = unique haplotypes (%), *A* = Mean number of alleles, *H* = Average gene diversity over loci, *UG* = unique multilocus genotypes (%), *H*_*E*_ = Expected Heterozygosity, *H*_*O*_ = Observed Heterozygosity, *P* = Polymorphic loci (%), *Average over all samples, individuals analysed independently from their geographic origin.

#### Genotypes

79 haplotypes were discovered from the analysis of the cpSSRs when every allele from each individual were combined. Among them, 38 were unique and 41 were shared among two or more individuals (up to 10 individuals). In contrast, 165 unique multilocus genotypes were found for the nuclear markers (90.2% of the individuals). Individuals WEST6, 7, 8 on the one hand and CORR2, 4, 6, 7, 8 on the other hand have the same genotypes. When the results are combined with the cpSSRs, CORR2 and 6, CORR4 and 7, and WEST7 and 8 have the same genotype, demonstrating that these individuals might be clonal.

### Genetic structure

*G*_*ST*_ was moderate for the cpSSR data (mean *G*_*ST*_ = 0.38, Table 2). *F*_*ST*_ calculated with the nuclear SSR data was between low and moderate depending on the locus (0.09-0.28, Table 3) and the *G*_*ST*_ was approximately twice as low (0.04-0.12) suggesting low differentiation. The ratio of pollen mediated/seed mediated gene flow was calculated according to Petit et al., 2005 [33]. The mean values of *G*_*ST*_ from Table 2 and Table 3 were taken to calculate the ratio, which was found to be equal to 6.8.

A third measure of differentiation was calculated in GenAlEx, Jost’s *D* (*D*_*est*_) [30]. Jost suggests that when using highly polymorphic markers to examine differentiation among populations, *G*_*ST*_ or its analogues should not be used because when diversity is high this measure will approach zero (no differentiation). These data support this, as SB85 was the least variable and SB38 was the most variable even though their *G*_*ST*_ is similar (0.04 against 0.06). *D*_*est*_ was very low for SB85 (0.002) and considerably higher for SB38 and SB194 (0.33 and 0.27). It was moderate for SB24 and SB93.

#### AMOVA

Two locus by locus AMOVA analyses per marker type were carried out in Arlequin using two distance measures: *F*_*ST*_ and *R*_*ST*_ (Table 5). From the cpSSR data, both analyses showed that the variation was mostly within populations (70% for the *F*_*ST*_ based AMOVA and 63% for the *R*_*ST*_ based AMOVA), the rest of the variation being among populations (Table 5 A and B). Genetic differentiation among populations was found to be moderate as the *F*_*ST*_ associated with both AMOVAs were significant at 0.304 and 0.371. The two AMOVAs computed with the nuclear data did not produce the same results. The first AMOVA based on *F*_*ST*_ shows that most of the variation was within individuals (68.9%, Table 5 C) while for the AMOVA based on the *R*_*ST*_ analysis, it shows that most of the variation was among individuals within populations (62.4%, Table 5 D). A negative variance component was found in Table 5 D, resulting in a negative *R*_*ST*_ which sometimes occurs because what is calculated is a covariance. It shows that there is an absence of genetic structure. It can also have a biological meaning. For instance, in dioecious organisms like *S. caprea*, genes from different populations can be more related to each other than genes from the same population.

**Table 5:** **Analyses of molecular variance for cpSSR and nuclear SSR data**

	**AMOVA (cpSSRs)**	**Sum of squares**	**Variance components**	**Explained variance %**	***F***_***ST***_
A) Based on *F*_*ST*_	Among sites	114	0.53	30.4	0.304***
	Within sites	192	1.21	69.6	
	Total	306	1.74	100	
B) Based on *R*_*ST*_	Among sites	493	2.46	37.1	0.371***
	Within sites	654	4.16	62.9	
	Total	1147	6.62	100	
	**AMOVA (nuclear SSRs)**	**Sum of squares**	**Variance components**	**Percentage variation**	***F***_***ST***_
C) Based on *F*_*ST*_	Among sites	85	0.15	8.3	0.083***
	Among individuals within sites	284	0.40	22.8	
	Within individuals	217	1.22	68.9	
	Total	586	1.77	100	
D) Based on *R*_*ST*_	Among sites	10229	−5.59	−1.8	−0.018^NS^
	Among individuals within sites	51350	193.97	62.4	
	Within individuals	20574	122.25	39.4	
	Total	82153	310.63	100	

***p-value over 1000 permutations < 0.0001; ^NS^non-significant *p*-value > 0.05.

#### IBD

For the cpSSR data, the first Mantel test among all individuals showed a slight pattern of isolation by distance, although the slope was nearly equal to zero (y = 0.0016x + 3.2762; R^2^ = 0.0051, *p* <0.0001). However, the 2^nd^ test with Slatkin linearized *F*_*ST*_ showed no IBD (y = 5.10^−5^x + 0.4624; R^2^ = 5.10^−5^, *p* = 0.426). For the nuclear SSR data, similar results to the cpSSR analysis were obtained (data not shown). The first test among all individuals was significant (y = 0.0036x + 8.961; R^2^ = 0.0046, *p* = 0.003) but the Mantel test based on population *F*_*ST*_ showed no significance (y = −0.6.10^−04^x + 0.1108; R^2^ = 0.0038, *p* = 0.266).

#### Bayesian clustering

**Figure 2:**
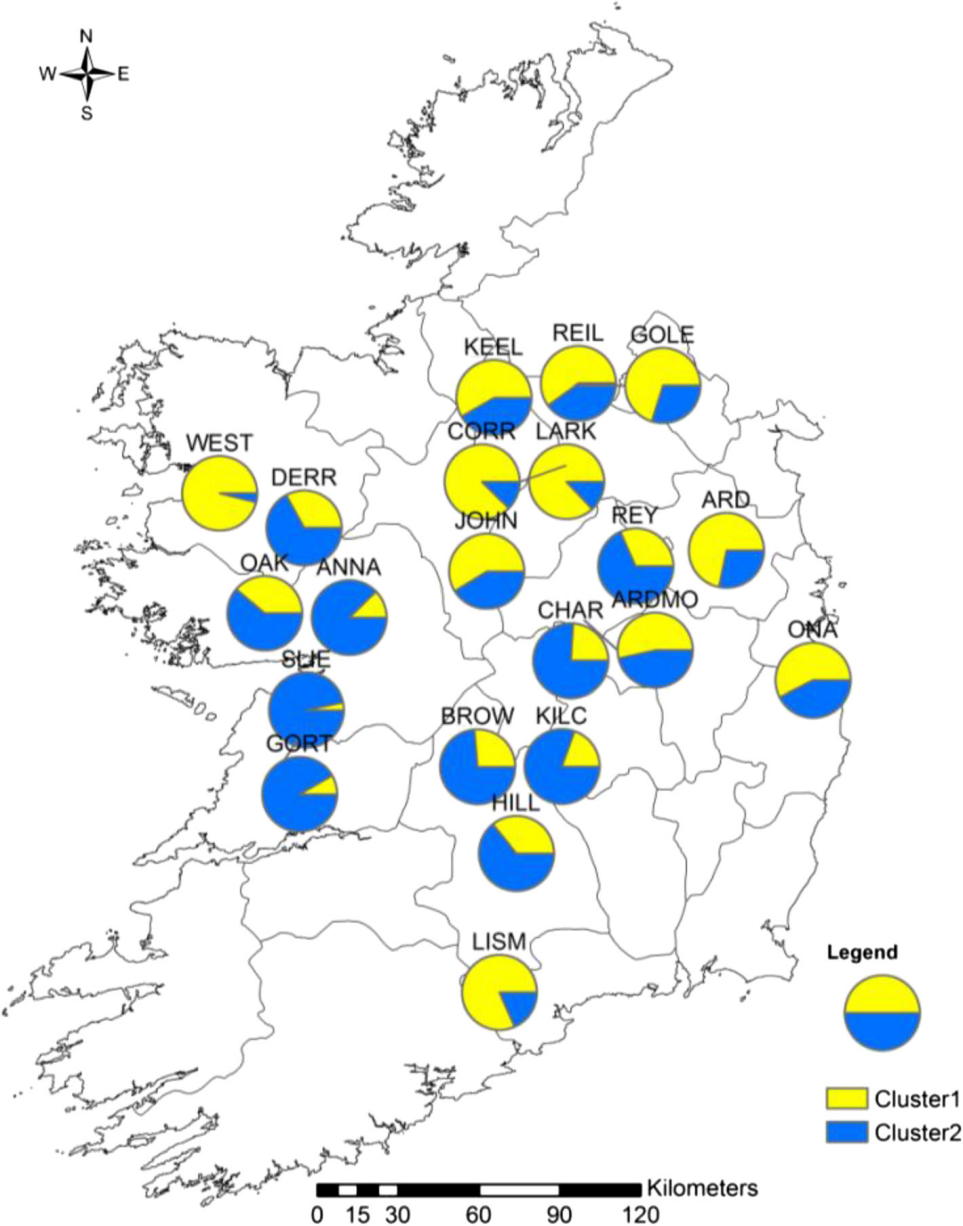
**Cluster identity of individuals within** ***S. caprea*** **populations obtained from STRUCTURE for the chloroplast SSR analysis.** Between 7 and 23 individuals per population were mapped.

**Figure 3:**
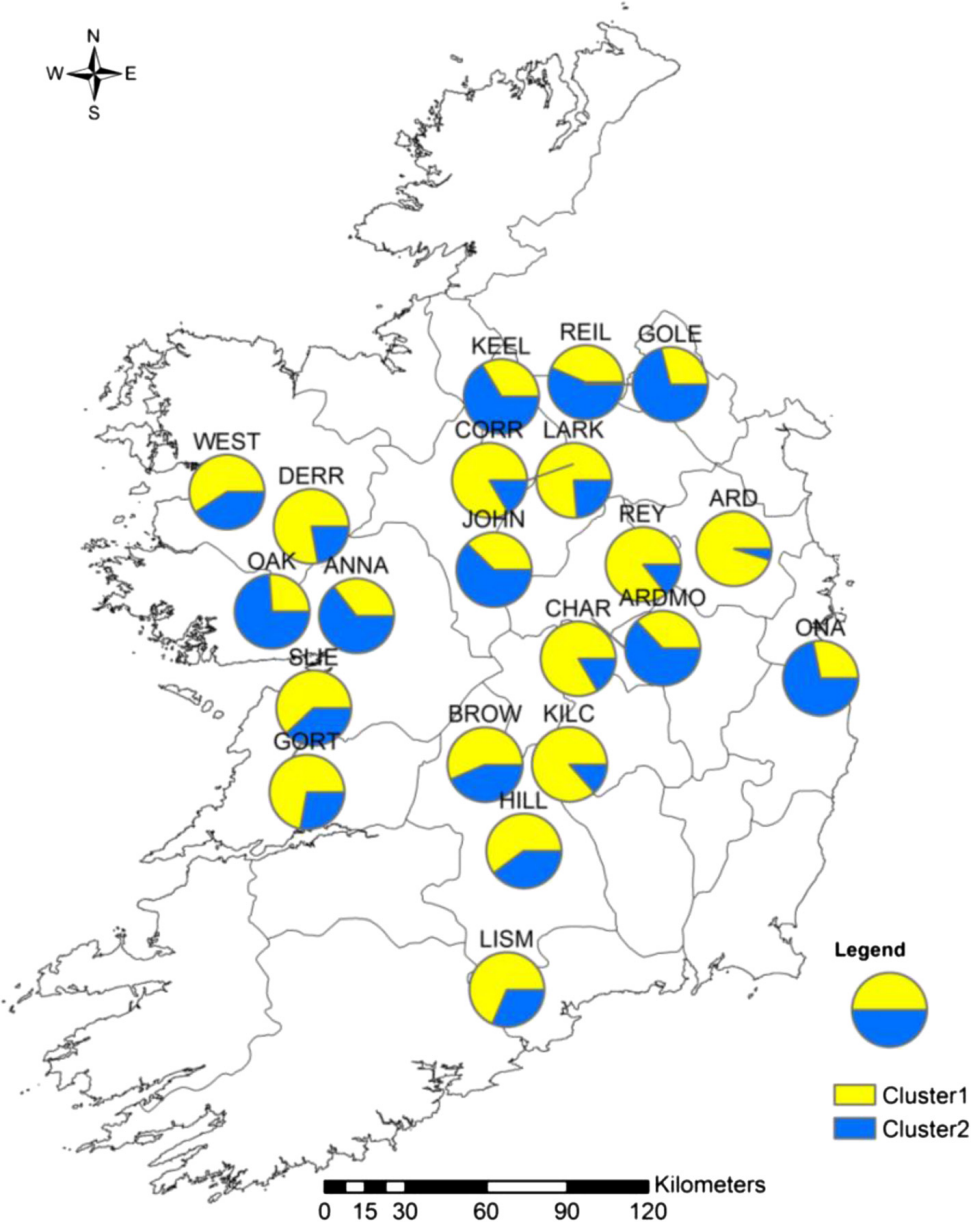
**Cluster identity of individuals within** ***S. caprea*** **populations obtained from STRUCTURE for the nuclear SSR analysis.** Between 7 and 23 individuals per population were mapped.

The clustering implemented within STRUCTURE software supported an optimal value of *K* to be *K* = 2 for both types of markers. The two clusters were mapped for each population (cpSSR: Figure 2, nuclear SSR: Figure 3). A slight geographic pattern of structure was detected especially in the cpSSR analysis. For instance, individuals mostly associated with cluster 2 were more common in the western populations and individuals mostly associated with cluster 1 in the eastern populations. Such structuring is consistent with the AMOVA results for cpSSRs. WEST had a different pattern from the other western populations. ANNA, SLIE and GORT had very similar patterns, and so too did LISM, LARK and CORR. For the nuclear SSR analysis, the OAK, ANNA, KEEL, REIL, GOLE, JOHN, ARDMO and ONA populations had a similar pattern while cluster 1 was more common in the other populations.

## Discussion

### Genetic diversity and gene flow

Every SSR marker primer pair successfully amplified the target DNA in *S. caprea*. The cpDNA markers were designed as universal markers in dicotyledonous angiosperms on tobacco [25] and this study has demonstrated their utility in *Salix caprea*. The same is true of the nuclear microsatellite markers applied in this study. They detected a high level of diversity and were useful for studies of population genetic structure. The nuclear SSR markers were designed for *Salix burjatica* [10] and have been shown to work well here on *S. caprea*. The allele size ranges found in this study are within the ranges found in Barker et al., 2003 [10] except for SB85, SB93 and SB199 where the size range was slightly different (Table 3).

Some of the CCMP alleles can be compared with those found for another microsatellite study on 24 populations of *S. caprea* sampled across Europe [15]. In both studies, CCMP7 was found to be monomorphic (135 bp against 133 bp, Table 2). CCMP10 had limited variation: 3 alleles were found (107, 109, 110 bp) in Palmé et al., 2003 [15] against 2 for our study (102 and 103 bp). Five alleles were found for CCMP2 in Palmé et al. [15] (206, 208-211 bp). Six alleles of nearly similar sizes were found in our study (210-215 bp). The fragment analysis method used was not the same, manual acrylamide gels were run in Palmé et al. [15] which could explain the differences.

All *S. caprea* individuals studied displayed a high level of cpDNA and nuclear DNA SSR allelic variation and a considerable number of genotypes were found within and among populations. This high allelic variation could be explained by the fact that *S. caprea* is an outcrossing species producing numerous very small seeds, bearing a tuft of long hairs encouraging wind dispersal [41]. Propagules could also have been moved deliberately or accidentally by, for example, grazers, birds or humans. We have detected numerous genotypes within and between *S. caprea* populations which would suggest that seed dispersal is high over Ireland. It is consistent with a study on natural populations of *S. viminalis* in the Czech Republic [42] that found that 92% of the individuals had unique multilocus genotypes with 38 nuclear microsatellites analysed. A few individuals were found to have both the same chloroplast and nuclear genotypes (CORR2 and CORR6, CORR4 and CORR7, WEST7 and WEST8). Microsatellite markers and especially the nuclear ones in this study are highly polymorphic, so it can be inferred that these individuals could be clonal. However, as *S. caprea* seems to be a species that is recalcitrant against natural vegetative regeneration [3], it is possible that these individuals are introgressed hybrids of *S. caprea*, probably with *S. cinerea* ssp. *oleifolia* (*S.* ×*reichardtii* Kern.), as this hybrid is frequent in Ireland [13] and it was noted during morphological examination in the Corratober (CORR) population when the samples were collected.

All populations showed relatively high values of observed heterozygosity (*H*_*O*_ = 0.41 for the nuclear SSRs) and gene diversity (*H* = 0.39 for the cpSSRs and *H* = 0.53 for the nuclear SSRs) which are comparable to other Salicaceae like *Populus tremuloides*, *S. purpurea*, *S. viminalis* or *S. daphnoides* [18,19,42,43]. Overall genetic variability for the samples studied, represented by Shannon’s information index values, was particularly high with an average of 1.48 for the cpSSRs and 1.35 for the nuclear SSRs. The high value of Shannon’s index represents the effectiveness of microsatellite loci to reveal the variation.

*Nm*, estimated gene flow from *F*_*ST*_ was 2.29 for the cpSSRs and 2.76 for the nuclear SSRs on average. In both cases *Nm* was superior to 1.0, which shows a constant gene flow between populations (i.e. at least one migrant per generation); therefore populations are expected to remain genetically stable over time [44]. *Nm* decreases with increasing *F*_*ST*_ because greater differentiation between populations corresponds to lower levels of gene flow [11]. For an outcrossing, dioecious species like *S. caprea* which is partly wind pollinated, gene flow is expected to be high between and within populations [45]. From our data, gene flow is expected to occur by pollen and a bit less through seeds. In fact, the ratio of pollen mediated/seed mediated gene flow was found to be approximately equal to 7. It indicates that gene flow via pollen is approximately 7 times higher than via seeds. It is not as high as the median of the ratio found in Petit et al., 2005 [33], which was based on 93 plant species and equal to 17. Seed dispersal in *S. caprea* appears to account for a large (roughly 13%) component of total gene flow.

### Population structuring

Genetic differentiation (*G*_*ST*_) of populations was pronounced for the cpSSRs (0.38 on average) but low for the nuclear microsatellites (0.07 on average). It is not completely unexpected as cpDNA is generally maternally inherited in angiosperms [46,47] and has therefore a smaller effective population size than nuclear DNA. Hence, genetic drift acts more intensively upon chloroplast than nuclear DNA, although pollen mediated and seed mediated gene flows were found to be nearly equal. Maternal inheritance also means that cpDNA is only dispersed through seeds. It implies that *G*_*ST*_ among populations is generally more pronounced for cpDNA than nuclear DNA. *G*_*ST*_ was found to be much lower for populations of *S. caprea* sampled across Europe for chloroplast DNA [15]. They have found a *G*_*ST*_ of 0.090 for PCR-RFLP markers and a *G*_*ST*_ of −0.017 for cpSSRs.

From the nuclear SSR analysis, low to moderate genetic differentiation between populations was discovered depending on the method used (*F*_*ST*_ = 0.08-0.16; *D*_*est*_ = 0.10; *G*_*ST*_ = 0.07). These values are higher than those estimated for natural populations of *S. viminalis* in the Czech Republic (*F*_*ST*_ of 0.05) based on 38 nuclear SSRs [42] but comparable to Bulgarian populations of *Fraxinus excelsior* trees (*F*_*ST*_ of 0.09) based on six nuclear SSRs [48].

AMOVA results for the cpSSR study showed that most of the variation was within populations but among population variation was moderate (30 to 37%). For the nuclear SSRs study, however, the among population genetic variation estimations were substantially lower and differed depending on the method used. Most of the variation was estimated to occur within individuals for the *F*_*ST*_ -AMOVA but mostly among individuals within sampling sites for the *R*_*ST*_ -AMOVA. These results are in accordance with other outcrossing woody species [49] and with other Salicaceae like *Populus nigra* or *S. viminalis* [42,50]. In another study on 16 populations of *P. nigra* across Europe, 90% of the genetic variation was found within populations for the microsatellite data used [51]. The results of these studies are based on *F*_*ST*_ only.

The tests for isolation by distance gave similar results for both the cpSSR and nuclear markers. A slight IBD was identified for the tests among all individuals on pairwise distances but no IBD was detected for the tests with the linearized *F*_*ST*_ among the sampled sites. This is in accordance with a study on *S. viminalis* in the Czech Republic [42]. The IBD tests were also in accordance with the Bayesian analysis of the possible structuring of the populations. This analysis identified two putative clusters for both analyses, but little obvious geographical pattern was detected for the clusters. From the cpSSR study, a slight North-East versus West structuring could be detected. It was especially visible for the two populations in the Burren, GORT and SLIE. These populations are nearly only clustered in cluster 2 which could be indicative of a limited gene flow through seeds with the other populations. Human activities in the Burren are perhaps lower than the other regions, possibly reducing the amount of artificial gene flow. The limited sub-structuring detected may also be influenced by eco-geographical factors such as rainfall, temperature and soil type. This structuring is not shown in the nuclear analysis, probably indicating a stronger pollen-mediated gene flow between populations. Our study, despite detecting some among population differentiation with the cpSSR markers, is largely consistent with a study on populations of *S. caprea* across Europe where an absence of geographical structure was found from the analysis of three cpSSRs and four PCR-RFLPs [15].

Sexual reproduction is inferred to be high for *S. caprea* within the sampling area and this is expected as *S. caprea* is recalcitrant to natural vegetative regeneration except for a few genotypes [3]. Our data, in which a high number of multilocus genotypes were unique to a single individual (90% for the nuclear markers) and *G*_*ST*_/*F*_*ST*_/*D*_*est*_ values were low especially for the nuclear SSRs, are consistent with outbreeding and indicate that there are no significant barriers for sexual reproduction and gene flow within Ireland over large geographic distances.

Both pollen-mediated and seed-mediated gene flow are high, some of the populations being 230 km apart from each other. Such a finding could simply be due to human intervention through seed trade, extensive planting or accidental transportation of both seeds and pollen. The absence of population structure could indicate the existence of one largely continuous population throughout Ireland. A parentage study has not been undertaken in our work but the results could also be consistent with long distance pollination. Parentage analyses were undertaken in *Populus trichocarpa* [52]. In a study site of >300 km^2^ (radius 10 km) in western Oregon, the mean pollination distance was 7.6 km, with many recorded matings over 10 km [52]. They could not estimate the maximum distance pollen could travel in *P. trichocarpa*, as the maximum within stand pollination distance simply reflects the maximum potential distances between trees within the study site, which was verified for other studies [53].

## Conclusion

This paper used chloroplast and nuclear microsatellites to examine the genetic diversity, the geographic population genetic structure and the extent of gene flow in natural populations of *S. caprea* across Ireland. New markers were tested and shown to be suitable for genetic characterization of *S. caprea*. High levels of allelic and genetic diversity were found with these markers, with within population variation accounting for the majority of the variation, and a high number of unique genotypes detected. Population structure and differentiation analyses, as well as IBD tests confirmed low levels of geographical structuring of variation but moderate differentiation was detected with the cpSSRs. However, gene flow through seeds and pollen was shown to be large. These results are of value for breeders wishing to exploit natural populations and foresters having to choose planting material. Further analysis should analyse the variation of Irish populations in relation to those found in Europe.
